# Supplementary material for: Mapping the evolution of fertility support policies in China: A content and instrumental analysis
Source: PLoS One. 2025 Oct 9;20(10):e0332137. doi: 10.1371/journal.pone.0332137 (PMC12510515; doi:10.1371/journal.pone.0332137)
Supplement: S1 Appendix — (ZIP) [file pone.0332137.s001.zip › S1 Appendix. 226 original policy documents/63-国务院办公厅关于印发完善促进消费体制机制实施方案(2018—2020年)的通知(FBM-CLI-2-322560).doc]

国务院办公厅关于印发完善促进消费体制机制实施方案(2018—2020年)的通知

**发布部门：** [国务院办公厅](https://www.pkulaw.com/law/chl?Aggs.IssueDepartment=601) [机构沿革](javascript:void(0))

**发文字号：**国办发〔2018〕93号

**发布日期：**2018.09.24

**实施日期：**2018.09.24

**时效性：** [现行有效](https://www.pkulaw.com/law/chl?Aggs.TimelinessDic=01)

**效力级别：** [国务院规范性文件](https://www.pkulaw.com/law/chl?Aggs.EffectivenessDic=XC0203)

**法规类别：** [机关工作综合规定](https://www.pkulaw.com/law/chl?Aggs.Category=00301)

国务院办公厅关于印发完善促进消费体制机制实施方案（2018-2020年）的通知
（国办发〔2018〕93号）

各省、自治区、直辖市人民政府，国务院各部委、各直属机构：
　　《完善促进消费体制机制实施方案（2018-2020年）》已经国务院同意，现印发给你们，请认真贯彻执行。

国务院办公厅
2018年9月24日

　　完善促进消费体制机制实施方案（2018-2020年）

　　消费是最终需求，促进消费对释放内需潜力、推动经济转型升级、保障和改善民生具有重要意义。为加快破解制约居民消费最直接、最突出、最迫切的体制机制障碍，增强消费对经济发展的基础性作用，根据《[中共中央 国务院关于完善促进消费体制机制进一步激发居民消费潜力的若干意见](https://www.pkulaw.com/chl/50dd2ac3d611cf7dbdfb.html)》，制定本实施方案。
　　**一、**进一步放宽服务消费领域市场准入
　　（一）旅游领域。制定出台海南建设国际旅游消费中心实施方案。及时总结59国外国人海南入境旅游免签政策实施效果，加强出入境安全措施建设，为进一步扩大免签范围创造条件。制定出台自驾车、旅居车营地建设相关规范。逐步放开中外合资旅行社从事旅游业务范围，加强与国际旅游组织的合作。制定出台邮轮旅游发展规划、游艇旅游发展指导意见。出台实施进一步促进乡村旅游提质升级的政策措施。推进生态航道建设，研究开发京杭运河具备条件航段的航运旅游功能，促进交通与旅游融合发展。理顺出租汽车、汽车租赁等个性化、差异化出行价格机制。鼓励发展租赁式公寓、民宿客栈等旅游短租服务。落实带薪休假制度，鼓励错峰休假和弹性作息。（国家发展改革委、文化和旅游部、外交部、海关总署、公安部、农业农村部、商务部、人力资源社会保障部、交通运输部按职责分工负责）
　　（二）文化领域。在文化服务领域开展行政审批标准化试点。推进经营性文化事业单位转企改制、公益性文化事业单位改革和国有文化企业公司制股份制改造。制定实施深化电影院线制改革方案，推动“互联网＋电影”业务创新，完善规范电影票网络销售及服务相关政策，促进点播影院业务规范发展。拓展数字影音、动漫游戏、网络文学等数字文化内容。完善游戏游艺设备分类，严格设备类型与内容准入。总结推广引导城乡居民扩大文化消费试点工作经验和有效模式。扩大文化文物单位文化创意产品开发试点范围。清晰界定文物的所有权、保管权和收藏权，完善文物合法流通交易体制机制。（中央宣传部、文化和旅游部、国家文物局、广电总局、国家发展改革委、财政部按职责分工负责）
　　（三）体育领域。加快制定赛事审批取消后的服务管理办法，建立体育、公安、卫生等多部门对商业性和群众性大型赛事活动联合“一站式”服务机制。修订彩票公益金资助开展全民健身赛事和活动有关办法，研究制定向社会力量购买全民健身赛事活动服务的办法。推进体育赛事制播分离，积极打造国家体育传播平台，引导有条件的地方电视台创办体育频道。打破大型国际体育赛事转播垄断，引入体育赛事转播竞争机制，按市场化原则建立体育赛事转播收益分配机制。积极培育冰雪运动、山地户外运动、水上运动、航空运动、汽车摩托车运动、电竞运动等体育消费新业态。支持海南打造国家体育旅游示范区，引入一批国际一流赛事。开展全民运动健身模范市（县）创建。（体育总局、财政部、国家发展改革委、广电总局按职责分工负责）
　　（四）健康领域。落实医疗卫生服务体系规划、医疗机构设置规划、大型医用设备配置规划，凡符合规划条件和准入资质的，不得以任何理由限制。合理放宽社会办医疗机构配置大型医用设备规划预留空间。实施社会办医疗机构跨部门联合审批。制定新型健康服务机构准入标准和监管办法。加快推动贫困县县医院远程医疗全覆盖，并推动向乡村延伸。适应“互联网＋医疗健康”发展，健全互联网诊疗收费政策，逐步将符合条件的互联网诊疗服务纳入医保支付范围。（国家卫生健康委、国家发展改革委、人力资源社会保障部、财政部、国家医保局、商务部按职责分工负责）
　　（五）养老领域。取消养老机构设立许可。建立养老机构分类管理制度，加快推进公办养老机构转制为企业或开展公建民营，建立健全养老领域公建民营相关规范，着力解决托底保障职能与公建民营不协调问题。编制实施国家积极应对人口老龄化中长期规划，支持各类市场主体增加养老服务供给。推动医养结合，研究出台医养结合机构服务和管理指南，深入开展长期护理保险试点。开展养老机构服务标准体系建设和养老机构服务质量专项行动。推动社区养老服务设施全覆盖。（民政部、国家卫生健康委、人力资源社会保障部、国家医保局、银保监会、国家发展改革委、商务部、市场监管总局按职责分工负责）
　　（六）家政领域。健全家政服务标准和服务规范，研究制定家政电商等新兴业态的服务标准和规范，开展家政服务标准化试点示范建设。强化家政服务从业人员岗前培训，育婴员、养老护理员等职业实行在岗继续培训制度。实行上岗前健康体检制度，家政服务从业人员上岗前按所从事家政服务类别进行体检。探索建立家政服务保险制度，鼓励家政服务从业人员投保人身意外伤害保险，支持商业保险机构开发家政服务雇主责任保险、职业责任保险、意外险等保险产品。支持家政服务知名品牌建设，引导家政企业做大做强。建立健全大中型城市与中西部地区国家级贫困县之间的家政服务劳务对接机制。（国家发展改革委、商务部、人力资源社会保障部、银保监会、市场监管总局按职责分工负责）
　　（七）教育培训领域。抓紧修订[民办教育促进法实施条例](https://www.pkulaw.com/chl/0eee9ad947e9fafabdfb.html)。开展产教融合建设试点工作，鼓励有条件的地区探索职业学校股份制改革试点。大力发展普惠性学前教育，鼓励地方政府通过购买服务、减免租金、派驻公办教师等方式引导和支持民办幼儿园提供普惠性服务。制定实施大力发展3岁以下婴幼儿照护服务的政策措施。实施好[关于规范校外培训机构发展的意见](https://www.pkulaw.com/chl/5d53d9312296111bbdfb.html)。鼓励有条件的地区引进境外优质高等教育、职业教育资源，举办高水平中外合作办学机构和项目，探索建立本科以上层次中外合作办学项目部省联合审批机制。（教育部、商务部、国家发展改革委按职责分工负责）

　　**二、**完善促进实物消费结构升级的政策体系
　　（八）大力发展住房租赁市场。总结推广住房租赁试点经验，加快研究建立住房租赁市场建设评估指标体系。发挥国有租赁企业对市场的引领、规范、激活和调控作用，支持专业化、机构化住房租赁企业发展。加快建设政府主导的住房租赁管理服务平台。鼓励有条件的城市结合实际探索发展共有产权住房，多渠道解决群众住房问题。加快出台城镇住房保障条例、住房租赁条例和住房销售管理条例。建立健全房地产信息发布机制，加强舆论引导，稳定市场预期。（住房城乡建设部、国家发展改革委按职责分工负责）
　　（九）促进汽车消费优化升级。继续实施新能源汽车车辆购置税优惠政策，完善新能源汽车积分管理制度，落实好[乘用车企业平均燃料消耗量与新能源汽车积分并行管理办法](https://www.pkulaw.com/chl/5dc9a479f5254019bdfb.html)，研究建立碳配额交易制度。完善新能源汽车充电设施标准规范，大力推动“互联网＋充电基础设施”，提高充电服务智能化水平。研究制定促进智能汽车创新发展的政策措施。实施[汽车销售管理办法](https://www.pkulaw.com/chl/2b56c372f2851837bdfb.html)，打破品牌授权单一模式，鼓励发展共享型、节约型、社会化的汽车流通体系。深入推进汽车平行进口试点。全面取消二手车限迁政策，便利二手车交易。修订[报废汽车回收管理办法](https://www.pkulaw.com/chl/9680ecba6ee1f918bdfb.html)。积极发展汽车赛事、旅游、文化、改装等相关产业，深挖汽车后市场潜力。综合运用发行城市停车场建设专项债券、调整完善车辆购置税分配政策等措施，加大停车设施建设资金支持力度。（商务部、工业和信息化部、国家发展改革委、交通运输部、财政部、税务总局、自然资源部、住房城乡建设部、海关总署、生态环境部按职责分工负责）
　　（十）发展壮大绿色消费。加大相关标准标识认证制度实施力度，完善政府采购制度，创新领跑者指标和相关技术标准的衔接机制。统一常用电子产品的电池、充电器标准。研究建立绿色产品消费积分制度。推动绿色流通发展，倡导流通环节减量包装、使用可降解包装。创建一批绿色商场，在继续做好绿色购物中心创建基础上，逐步向超市、专业店等业态延伸，引导流通企业增设绿色产品专区，扩大绿色产品销售，积极发挥绿色商场在促进绿色循环消费方面的示范作用。（国家发展改革委、生态环境部、商务部、市场监管总局按职责分工负责）
　　（十一）进一步扩大和升级信息消费。加大网络提速降费力度。加快推进第五代移动通信（5G）技术商用。支持企业加大技术研发投入，突破核心技术，带动产品创新，提升智能手机、计算机等产品中高端供给体系质量。支持可穿戴设备、消费级无人机、智能服务机器人等产品创新和产业化升级。利用物联网、大数据、云计算、人工智能等技术推动各类应用电子产品智能化升级。创新发展生活类信息消费，重点发展面向社区生活的线上线下融合服务、面向文化娱乐的数字创意内容和服务、面向便捷出行的交通旅游服务。推进网络游戏转型升级，规范网络游戏研发出版运营。培育形成一批拥有较强实力的数字创新企业。建立健全公共数据资源开放共享体系。（中央网信办、中央宣传部、工业和信息化部、国家发展改革委、科技部、文化和旅游部、广电总局按职责分工负责）
　　（十二）推动传统商贸创新发展。高标准布局建设具有国际影响力的大型消费商圈，完善“互联网＋”消费生态体系，鼓励建设“智慧商店”、“智慧商圈”。支持具备条件的城市重点培育一批具有国际国内领先水平的高品位步行街，促进线上线下互动、服务体验融合、商旅文体协同、购物体验结合。提升城市配送车辆通行管理水平。优化商贸物流设施空间布局，大力发展便利店、社区菜店等社区商业。建设培育特色商贸小镇。推进经济型酒店连锁经营，鼓励发展各类生态、文化主题酒店和特色化、中小型家庭旅馆。（商务部、国家发展改革委按职责分工负责）

　　**三、**加快推进重点领域产品和服务标准建设
　　（十三）深入实施“同线同标同质”工程。将内外销产品“同线同标同质”工程实施范围逐步由食品农产品领域向消费品等其他领域拓展。（市场监管总局负责）
　　（十四）加强消费产品和服务标准制定。积极开展质量提升行动，加强质量基础设施建设与应用，开展国家质量基础设施协同服务及应用。开展新业态、新消费以及服务消费质量监测专项行动，瞄准智能产品、重点服务消费等领域，综合采取风险监测、质量分析、标准领航等措施提升质量水平。修订提高生鲜乳、灭菌乳等国家标准，建立全过程质量追溯体系。积极开展体育、旅游、家政、养老等服务消费领域和以信息技术为支撑的消费新业态新模式的国家标准制订工作，选择部分服务业探索开展服务标准准入制试点。开展健康、教育、体育、金融、电商等领域服务认证。完善绿色产品标准体系，重点加快制修订能源、涉挥发性有机物等产品相关标准，抓好标准实施。有序推动强制性国家标准立项工作，整合精简现行强制性国家标准、行业标准和地方标准。（市场监管总局、工业和信息化部、国家发展改革委、商务部、国家卫生健康委、生态环境部按职责分工负责）
　　（十五）打造有影响力的中国品牌。选择部分重点行业和重点产品，推动制定国内领先、国际一流的企业标准和行业标准，创建一批有世界影响力的中国品牌。（市场监管总局、工业和信息化部、国家发展改革委、住房城乡建设部、商务部按职责分工负责）

　　**四、**建立健全消费领域信用体系
　　（十六）完善消费领域信用信息共享共用机制。依托全国信用信息共享平台，聚焦重点消费品和旅游、家政、养老、健康等服务消费，进一步归集市场监管、税务、海关等领域信用信息，建立健全企业信用档案和人员档案数据库。落实企业的信息共享共用主体责任，督促企业加大基本信息数据收集力度，实现产品生产信息和质量追溯信息互联互通，方便消费者集中查询。出台实施商务领域诚信体系建设指导意见。（国家发展改革委、商务部、人民银行、海关总署、税务总局、市场监管总局按职责分工负责）
　　（十七）加强信用信息公开。强化“信用中国”网站公开功能，逐步实现行政许可、行政处罚、产品抽检结果等不涉及个人隐私、企业和国家秘密的信息向社会全部公开，为公众提供信用信息“一站式”查询和消费预警提示服务。建立重大信息公告和违法违规记录公示制度。（国家发展改革委、市场监管总局、商务部按职责分工负责）
　　（十八）健全守信激励和失信惩戒机制。加快建立守信“红名单”和失信“黑名单”及管理办法，实施守信联合激励和失信联合惩戒措施。在部分地区试点建立失信企业惩罚性赔偿制度，在总结评估的基础上逐步扩大试点范围。建立完善信用分级分类监管机制，根据企业信用风险等级合理配置和调度监管资源。（国家发展改革委、市场监管总局、商务部按职责分工负责）
　　（十九）推进消费者维权机制改革。开展放心消费创建工作。加强金融消费维权，强化监督管理和社会监督，约束引导银行业和保险业金融机构诚信服务、规范经营，不断提升金融消费满意度。加强电商消费维权，针对电商销售重要时间节点，加强与电商平台、消费者协会等的协作，实现维权、处罚等信息互联互通。严厉打击假冒伪劣和虚假广告宣传。推动加快个人信息保护法立法进程，完善消费者个人隐私、消费数据保护法律法规，强化零售商、服务提供商、电信运营商、互联网平台经营者的主体责任，加大对侵犯消费者隐私权行为的打击惩戒力度。（市场监管总局、人民银行、银保监会按职责分工负责）
　　（二十）加强重要产品质量追溯体系建设。统筹规划全国重要产品追溯体系建设，提高重要产品生产管理信息化、标准化、集约化水平，健全追溯大数据应用机制，逐步形成全国追溯数据统一共享交换机制，初步实现部门、地区和企业追溯体系互通共享。完善食品药品等重要消费品召回制度。扩大产品国家监督抽查覆盖面，增加抽查频次，加强联合监督执法。（商务部、市场监管总局、工业和信息化部、农业农村部按职责分工负责）

　　**五、**优化促进居民消费的配套保障
　　（二十一）完善有利于促进居民消费的财税支持措施。推动消费税立法。制定出台新[个人所得税法](https://www.pkulaw.com/chl/d74fa423af05cffdbdfb.html)相关配套制度和措施，明确专项附加扣除的具体范围、标准和操作办法。积极开展个人税收递延型商业养老保险试点工作。充分利用现有资金渠道对服务消费予以支持。（财政部、税务总局、银保监会、国家发展改革委、商务部按职责分工负责）
　　（二十二）进一步提升金融服务质效。在风险可控、商业可持续、保持居民合理杠杆水平的前提下，加快消费信贷管理模式和产品创新，加大对重点消费领域的支持力度，不断提升消费金融服务的质量和效率。引导商业保险机构加大产品创新力度，开发有针对性的保险产品。（人民银行、银保监会按职责分工负责）
　　（二十三）深化收入分配制度改革。进一步深化城乡居民增收综合配套政策试点、专项激励计划和收入监测试点。加快建立全国统一规范的企业薪酬调查和信息发布制度。加强和改进对企业工资分配的宏观指导。推进国有企业工资决定机制改革。完善机关事业单位工资和津补贴制度。合理确定社会救助、抚恤优待等标准。探索开展支出型贫困家庭救助工作。（人力资源社会保障部、财政部、民政部、国家发展改革委按职责分工负责）

　　**六、**加强消费宣传推介和信息引导
　　（二十四）加强消费领域统计监测。研究制定服务消费和消费新业态新模式的统计分类，完善旅游、文化、体育、健康、养老、家政、教育培训、广播电视等重点领域服务消费统计监测，研究建立较为全面反映服务消费发展水平的统计指标体系。建立并推行绿色产品市场占有率统计报表制度。健全消费政策评估机制，委托第三方机构开展重大消费政策评估。（国家统计局、商务部、生态环境部、市场监管总局、国家发展改革委按职责分工负责）
　　（二十五）加强消费领域大数据应用。依托国家数据共享交换平台体系等基础设施资源，加快推动各部门、各地区消费领域大数据应用并实现互联互通。推动社会组织、电商企业等建设相关领域大数据库，支持专业化大数据服务企业发展。加强政府与社会合作，建立消费领域大数据分析常态化机制，提升大数据运用能力。（国家发展改革委、工业和信息化部按职责分工负责）
　　（二十六）认真做好消费宣传引导工作。在重大消费政策出台前后，通过组织召开专题发布会、专家解读等方式，正确引导社会预期。定期组织召开消费形势或相关消费领域专题信息发布会，及时发布消费发展信息、增强社会信心。加强和改进新闻媒体关于消费文化的宣传报道。组织编制年度居民消费发展报告。倡导绿色消费理念，纳入全国节能宣传周、全国科普活动周、全国低碳日、世界环境日等主题宣传教育活动，通过新闻媒体、网络媒体开展公益宣传，营造绿色消费良好社会氛围。（中央宣传部、市场监管总局、广电总局、生态环境部、国家发展改革委、人民银行按职责分工负责）

© 北大法宝：（[www.pkulaw.com](https://www.pkulaw.com/)) 专业提供法律信息、法学知识和法律软件领域各类解决方案。北大法宝为您提供丰富的参考资料，正式引用法规条文时请与标准文本核对。
欢迎查看所有[产品和服务](https://www.pkulaw.com/helps/2.html)。法宝快讯：[如何快速找到您需要的检索结果？](https://www.pkulaw.com/helps/70.html)[法宝](https://www.pkulaw.com/helps/69.html)V6有何新特色？

扫描二维码阅读原文

**原文链接：**<https://www.pkulaw.com/chl/0bb7ae1ed7bbc06bbdfb.html>
